# The Effect of Child Quantity and Education on Parents' Well-Being in Vietnam

**DOI:** 10.3389/fpubh.2021.706279

**Published:** 2021-08-25

**Authors:** Linh Hoang Vu, Tung Duc Phung

**Affiliations:** ^1^Vietnam National University-Vietnam Japan University, Master Program of Public Policy, Hanoi, Vietnam; ^2^IPAG Business School, Paris, France; ^3^Mekong Development Research Institute, Hanoi, Vietnam

**Keywords:** family size, child education, older people's health, well-being, Vietnam

## Abstract

**Background:** Vietnam currently has a rapidly aging population, while formal social protection has only covered a small fraction of older people. Therefore, many older people with insufficient income or poor health must rely on their children's support.

**Method:** This study uses the Vietnam National Aging Survey 2011 to determine whether the quality of children's education/employment and the number of children in a family impact older people's life satisfaction and health.

**Results:** We find that the number of children has no effect on parents' life satisfaction but is adversely related to parents' health across a range of physical and mental health measures. In contrast, children's education has beneficial impacts on the well-being of elderly parents. Parents with better-educated children are more satisfied with their lives and report better health and fewer illness issues. Among other factors, income and wealth are strong and consistent predictors of older people's life satisfaction and self-perceived physical and mental health.

**Conclusion:** To the best of our knowledge, this is the first study to explore the relationship between quantity and quality of children and the well-being of elderly parents. Our results show that the number of children has an adverse effect on older people's self-reported health and life satisfaction in Vietnam. Meanwhile, parental health and life satisfaction are significantly related to children's education. The findings of this study provide several practical implications. Most importantly, investment in education for children will have long-lasting impacts on the health and well-being of elderly adults. In addition, our paper indicates that the government program for reducing fertility has contributed to the longer-term health of people.

## Introduction

For governments and scholars in developing countries, a priority is protecting older people in a society where social security is not well-developed. Data from the World Bank indicated that social insurance coverage in low-income and lower-middle-income countries was just 2.3 and 12.9%, respectively, compared to 39.7% in high-income countries during 1998–2014 (1). Furthermore, aging populations have become a global phenomenon, with Vietnam being one of the countries with the fastest increase in its elderly population (2). As of 2019, around 7.8% of the Vietnamese population, or approximately seven million, were 65 years old or older (3). The number of people aged 65 and older is projected to triple to 18.4 million by 2040, accounting for 17% of the population. The elderly dependency ratio in Vietnam (the percentage of people 65+ relative to the working-age population) is expected to almost triple from around 10% presently to 26% by 2040 (2).

The rapid increase in the aged population in Vietnam poses new challenges for policymakers, particularly in healthcare and social protection systems. The current official pension system remains low, at only around 22% of the labor force, and this system faces significant sustainability challenges that will increase as aging accelerates. Elderly parents in Vietnam still rely heavily on their children to care for them when they are old. Vietnam is also profoundly influenced by Confucian culture, with the belief that children are responsible for taking care of their parents in old age; therefore, “the more children, the greater prosperity.” While having more children could be considered a kind of insurance for parents in their old age, the quality of children as reflected by their social status, prosperity, and educational achievement can play a significant role in elderly parents' well-being. The relationship between child quantity and quality and a parent's well-being is an important topic to study. In the next section, we review the current literature related to the issue.

### Quantity-Quality Tradeoff

Since the seminal papers by Becker and Lewis (4) and Becker and Tomes (5), many studies have focused on the tradeoff between the quantity and quality of children. Empirical results are mixed. Studies of developed countries have often confirmed a negative relationship between family size and children's educational outcomes (6–8), while those in developing countries have reported more ambiguous effects (9–15).

### Children and Parental Life Satisfaction

The link between children and parents' life satisfaction is a topic of interest in developed countries. Most previous studies find adverse or null effects of children on parents' life satisfaction or life satisfaction (16, 17). Using data from the World Database of Happiness for over 100 countries, Blanchflower (18) reports that well-being is lower among adults with children. However, Angeles (19) finds a large, positive effect of the number of children on parents' life satisfaction. Still, the result is contingent on the individual's characteristics, as children make married people better off, while most unmarried individuals appear to be worse off with children. Cetre et al. (20) find that the association between children and subjective well-being is positive only in developed countries and for those who become parents after 30 and have a higher income. Meanwhile, Ugur (21) highlights the positive impact of having children on parents' life satisfaction; however, the effect has been substantially diluted over time in Europe.

Also, there are several studies on the effects of the number of children on elderly parents' life satisfaction, mostly in China. Again, the findings are mixed. Gao and Qu (22) indicate that more children can enhance elderly parents' subjective well-being (SWB), measured by either life satisfaction or depressed mood. In contrast, Shi (23) does not find that more children bring increased happiness to elderly parents.

### Quantity/Quality of Children and Parents' Health

Another strand of literature that we draw on is research on the effects of quantity and quality of children on elderly parents' health. Shi (23) and Islam and Smyth (24) find a negative relationship between the number of children and an elderly parent's health. Diaz-Venegas et al. (25) find that having fewer children is associated with lower chronic health conditions for the parents aged 50 plus in Mexico. Meanwhile, Hank (26) finds no differences in mental health linked with the number of children for middle-aged parents in Germany. Kruk and Reinhold (27) find a negative effect of the number of children on the mental health of older women but not older men in Europe, a result in contrast to Buber and Engelhard (28), who state that childless men had higher levels of depression, yet for women, the number of children had no effect on depression in Europe.

Regardless of the number of children, child quality can affect parents through several channels. First, children with higher educational attainment can provide better health knowledge to their parents (29, 30). Older people need to receive updated health knowledge and proper health care due to potential cognitive and functional decline in their old age. Educated children may provide their parents with better health-related advice and are more likely to supply their parents with high-quality care (31, 32). Second, better-educated children are often associated with better income; therefore, they can improve parental health and quality of life by providing financial support (33). Studies indicate that elderly parents receive monetary support from their adult children (34–37). Educated children are also more likely to purchase health care services or health insurance for their parents than less educated children (38).

Using longitudinal data from Mexico, Yahirun et al. (39) find that children's education positively impacts parental lifespan. Ludborg and Majlesi (40) suggest that better female schooling increases parents' survival in Switzerland, especially fathers' survival. They also indicate that female education's impact on fathers' longevity is larger for those from low socioeconomic backgrounds. Ma (33) demonstrates that more years of schooling for children results in better cognitive function and increased survival expectations for elderly parents in China.

While previous studies examined the linkage between child characteristics and parents' outcomes, to the best of our knowledge, there have been no studies that explore the effects of both quantity and quality of children on parents' health and well-being. This paper makes contributions to several aspects. First, it is the first study that examines the effect of children's quantity and quality on both the health and life satisfaction of elderly parents. As there may be a tradeoff between child quantity and quality, parents' decisions on the number of children they have can be influenced by weighting the expected benefits of fertility choice and investment in their children on their well-being in their old age. Thus, our study contributes to the evidence about the relationship between the decision on child quantity/quality and parental well-being. Second, in contrast to the previously mostly descriptive literature, we focus on identifying the causal effect of the number of children on parents' long-term health and well-being using the instrumental variable (IV) method. Third, it contributes to the empirical evidence on the impacts of children on parents' well-being in a populous middle-income country, for which there have been few analyses. As a middle-income country with rapid demographic change, Vietnam provides a test case since it has a rapidly aging population and a low fertility rate. There have been several studies on the determinants of life satisfaction and health among older people in Vietnam. For example, Tran et al. (41) suggest that some religious affiliations such as Buddhism and Caodaism are negatively related to happiness, while the relationship does not hold for Christians. Tran and Vu (42) find that housing satisfaction has a strongly positive impact on the life satisfaction of older people. Giang et al. (43) mention factors associated with depression among older people, including experiencing domestic violence, lack of money for daily living expenses, and living alone. However, the relationship between family size and parents' life satisfaction and health in Vietnam has not been examined. Finally, our results help evaluate Vietnam's two-child family planning policy.

Using data from the Vietnam National Aging Survey 2011, we find no evidence for a relationship between the number of children and parents' life satisfaction in their old ages. However, the number of children is negatively related to the parent's physical and mental health across several measures. Furthermore, there is a significant impact of the quality of children on the well-being of older people. In particular, higher education levels in children are associated with better life satisfaction, better health status, and less illness among older people.

## Method

### Data and Variables

The data were taken from the Vietnam National Aging Survey 2011, conducted by the Institute of Social and Medical Studies in October and November 2011. This unique survey on older people in Vietnam covered 4,007 individuals aged 50 years and above in 6 regions and 12 provinces in Vietnam. The questionnaire consisted of detailed information on older people, including their demographic backgrounds, family information, health status, work, income, and life satisfaction. Individual-level data include demographics, socioeconomic and health status. The data are considered nationally representative.

We used the following measures for parental well-being as dependent variables: (i) self-perceived well-being; (ii) self-rated physical health; (iii) illness status; (iv) limitations in activities of daily living (ADL); and (v) mental health.

- Self-perceived well-being. We used two indicators: relationship with family members and overall life satisfaction. The answers cover a five-point scale from *very dissatisfied* to *very satisfied*.
- Self-perceived health status of parents. This variable is measured based on a five-point rating scale ranging from (1) very good to (5) very poor.
- Illness status. This variable is calculated from 16 variables indicating whether the individual has experienced one of the following health problems during the last 30 days: headache, dizziness, vomiting, diarrhea, skin issues, chest pain, joint pain, fever, back pain, trembling hands, stomachache, breathing problems, coughing, loss of bladder control, feeling weak, and constipation. The scale ranges from 0 to 16.
- Limitations in daily living activities (ADL): The ADL variable measures older people's difficulty in doing the following activities: eating, getting dressed or undressed, bathing, getting up when lying down, and using the toilet. For each activity, the individual receives a score of one if they have difficulty doing that activity and zero otherwise. The total score is obtained by adding together all the scores for each activity. The maximum score is 5.
- Mental health issues: This variable indicates whether the individual has experienced the following symptom during the last 30 days: losing appetite, feeling sad or depressed, experiencing difficulty in sleeping, and feeling lonely. The total score for mental health is obtained similarly to the ADL score. The maximum score is 4.

In previous literature [e.g., (24, 25, 44)], explanatory variables often include parental characteristics such as age, sex, marital status, education, living arrangement, and income. Similarly, we use parent characteristics for the explanatory variables, including their age, gender, marital status, working status, living arrangement (e.g., with a son or daughter), ethnicity, religion, education, and personal income. As wealthier parents are more likely to invest in their children's human capital, we use “good housing” and “house areas” (in square meters) to control the wealth effect. Especially, “good housing” indicates if the family lives in a permanent house rather than a semi-permanent or a temporary one. In addition, the key explanatory variables of interest are the number of children and the child education variable. The number of children refers to the number of all biological living children. The child education variable is measured by the level of education of the most educated child or children in the family.

Table 1 displays the descriptive statistics. After dropping observations with missing values, the sample includes 3,603 individuals. On average, an older person has 4.4 children. Child quality is reflected in the highest level of education among the children. In 35% of the sample, the highest educational level among the children is vocational or University degree, while in 28% of the sample, the highest level of education among children is upper secondary education.

**Table 1:** Descriptive statistics.

	**Mean**	**Standard deviation**	**Min**	**Max**
**Dependent variables**				
Relationship	3.96	0.73	1	5
Happiness	3.69	0.85	1	5
Physical health	2.34	0.73	1	5
Illness	6.11	3.39	0	16
Mental health	1.88	1.35	0	4
ADL	0.70	1.30	0	5
**Independent variables**				
Number of children	4.38	2.12	1	14
Living with a son (1 = Yes, 0 = No)	0.55	0.50	0	1
Living with a daughter (1 = Yes, 0 = No)	0.28	0.45	0	1
**Child quality** (1 = Yes, 0 = No)				
Primary education	0.08	0.28	0	1
Lower secondary education	0.25	0.43	0	1
Upper secondary education	0.28	0.45	0	1
Vocational training or above	0.35	0.48	0	1
**Parent's characteristics**				
Age	66.00	11.30	50	100
Age at birth of the first child	25.57	5.37	15	55
Male (1 = Yes, 0 = No)	0.43	0.50	0	1
Married (1 = Yes, 0 = No)	0.70	0.46	0	1
Not working (1 = Not working, 0 = Working)	0.46	0.50	0	1
Kinh (majority) ethnicity (1 = Yes, 0 = No)	0.90	0.30	0	1
Religious (1 = Yes, 0 = No)	0.81	0.40	0	1
Primary education (1 = Yes, 0 = No)	0.18	0.38	0	1
Lower secondary education (1 = Yes, 0 = No)	0.22	0.42	0	1
Upper secondary or above (1 = Yes, 0 = No)	0.16	0.37	0	1
Log of income	1.15	0.27	1	2
**Housing characteristics**				
Log of house area (in m^2^)	4.11	0.61	2	6
Permanent house (1 = Yes, 0 = No)	0.32	0.47	0	1

### Empirical Strategy

To investigate the impact of quantity and quality of children on elderly parents aged 65 or above, we used the following model:

(1) $$\begin{array}{l} Y_{ij}=\alpha_{1}+\alpha_{2}N_{ij}+\alpha_{3}D_{ij}+\alpha_{3}X_{ij}+\lambda_{\text{j}}+\varepsilon_{\text{i}}. \end{array}$$

*Y*_*i*_ is the vector of well-being outcomes of parent *i*, living in district *j*, including such dimensions as relationship satisfaction, overall satisfaction, physical health status, illness status, mental health, and the ADL variable; λ_j_ is district fixed-effects.

*N*_*i*_ is the number of children of parent *i* in district *j*, and *D*_*ij*_ is the quality of education of the children of parent *i* in district *j*. Following Ma (33), we use the highest educated child's education level to proxy for children's quality. Thus, *D*_*ij*_ is measured by the highest level of education of children of parent *i*.

*X*_*ij*_ is a vector of control variables, reflecting the characteristics of parent *i* in district *j*.

#### Endogeneity Issues

Estimating the effect of child quantity on parents' health status and life satisfaction is not straightforward because of the potential endogeneity of the number of children. Broadly defined, endogeneity refers to situations in which an explanatory variable is correlated with the error term. In this study, unobserved attributes that are correlated with the household's fertility decisions are likely to be correlated with a parent's health status and life satisfaction. For example, both maternal and paternal health status may affect the number of children they have. Besides, endogeneity may occur due to measurement error, omitted variables, or simultaneity. In the presence of endogeneity, OLS can produce biased and inconsistent parameter estimates. The use of instrumental variables (IVs) is a common technique for addressing endogeneity issues (45). Yet, it is challenging to find a relevant and strong instrument as the inclusion of bad instruments can lead to biased estimates of the explanatory variables.

A popular instrument of addressing the endogeneity of the fertility decision is to use twins' natural occurrence to isolate the causal effect of family size (10, 46). Other approaches in constructing instrument variables for family size include parental preferences for a mixed-sex sibling composition (47, 48) and gender of the first child in a country with a strong son preference (13, 14). Several studies in China employed some measures of the enforceability or relaxation of the “one-child policy” as instrument variables (12, 49).

However, using twins to assess the number of children requires a massive dataset as twins in the population are rare. As our dataset was not very large, we could not use twins as an instrumental variable for fertility. We tried with both the mixed-sex sibling composition and the gender of the first child. However, both instrumental variables failed both the weak identification test and the under-identification test for instruments.

Our instrumental variable is based on the exogenous variation in family size caused by Vietnam's family planning policy launched in 1988 (50). Specifically, our instrumental variable is based on the “two-child” rule, which allowed families to have at most two children. Launched in 1988, the policy was applied to all Kinh (majority) ethnicity individuals, with exemptions for ethnic minority people. We use a time variable *before88*, assigning a value of one if the family already had at least two children by 1988. The instrument variable is the interaction of the time variable and the ethnic variable: before88^*^Kinh.

## Results

We regress the impact of the quantity and quality of children on elderly parents' quality of life using both the OLS and the IV method. We use the STATA 16 software and the *ivreg2* module (51). All standard errors are clustered at the commune level. The instrument passes both the under-identification and the weak instrument test. Furthermore, the first-stage regression results of the IV method are presented in Table A1 of the Appendix. The first-stage regression results indicate that the number of children is strongly correlated with the explanatory variables. The F-statistics of the first-stage regression is 175.7, far exceeding the rule-of-thumb of 10 for the weak instrument test proposed by Staiger and Stock (52).

The second condition to have a valid instrument is that it satisfies the exclusion restriction. In our case, this means that the family-planning policy only affects parental health and happiness through an increase in the number of children. This condition cannot be tested empirically. However, we did an exercise to check if the instrument possibly violated the exclusion restriction. At the first-stage regression, we instrumented fertility as usual. At the second stage, we added the instrument variable directly in the model to examine whether the instrument has an independent effect on parental health and life satisfaction. The results indicate that there is no direct effect of the family-planning policy on parental health and life satisfaction. Therefore, we conclude that our instrument is valid.

Tables 2, 3 report the effects of child quantity and quality on older people's life satisfaction and health.

**Table 2:** Effects on elderly parents' life satisfaction.

	**(1)**	**(2)**	**(3)**	**(4)**
	**OLS**		**IV**	
	**Relationship satisfaction**	**Overall satisfaction**	**Relationship satisfaction**	**Overall satisfaction**
**Child quantity and quality**				
Number of children	0.001	−0.008	−0.049	−0.025
	(0.009)	(0.009)	(0.037)	(0.040)
Primary school	0.015	0.028	0.029	0.033
	(0.082)	(0.094)	(0.079)	(0.094)
Lower secondary	0.127^*^	0.088	0.157^**^	0.098
	(0.077)	(0.092)	(0.077)	(0.095)
Upper secondary	0.203^***^	0.215^**^	0.243^***^	0.229^**^
	(0.075)	(0.098)	(0.077)	(0.104)
Vocational training or higher	0.235^***^	0.298^***^	0.283^***^	0.315^***^
	(0.079)	(0.091)	(0.080)	(0.100)
**Parent characteristics**				
Living with a son	−0.050^*^	−0.021	−0.022	−0.011
	(0.026)	(0.033)	(0.034)	(0.040)
Living with a daughter	−0.014	−0.014	0.013	−0.005
	(0.031)	(0.034)	(0.039)	(0.045)
Age	−0.014	0.012	−0.001	0.017
	(0.014)	(0.016)	(0.017)	(0.020)
Age at birth of first child	−0.000	−0.006^**^	−0.007	−0.009
	(0.003)	(0.003)	(0.006)	(0.006)
Male	0.084^***^	0.033	0.118^***^	0.045
	(0.030)	(0.029)	(0.033)	(0.038)
Married	0.080^**^	0.138^***^	0.111^***^	0.149^***^
	(0.033)	(0.039)	(0.043)	(0.047)
Not working	0.036	−0.042	0.025	−0.046
	(0.030)	(0.037)	(0.030)	(0.039)
Kinh (majority)	0.019	0.009	0.000	0.002
	(0.049)	(0.047)	(0.050)	(0.049)
Religion	0.026	0.031	0.018	0.029
	(0.027)	(0.037)	(0.029)	(0.037)
Primary	−0.039	0.029	−0.054	0.024
	(0.040)	(0.043)	(0.040)	(0.045)
Lower secondary	0.070^*^	0.099^**^	0.035	0.087
	(0.035)	(0.046)	(0.042)	(0.054)
Upper secondary	0.032	0.017	−0.024	−0.003
	(0.044)	(0.049)	(0.059)	(0.068)
Income (log)	0.186^***^	0.458^***^	0.157^**^	0.448^***^
	(0.058)	(0.070)	(0.062)	(0.077)
House area (log)	0.034	0.068^**^	0.040^*^	0.071^**^
	(0.022)	(0.031)	(0.023)	(0.031)
Good housing	0.045	0.121^***^	0.023	0.114^***^
	(0.031)	(0.039)	(0.033)	(0.042)
Constant	3.884^***^	2.053^***^	3.658^***^	1.972^***^
	(0.468)	(0.574)	(0.483)	(0.599)
*N*	3,404	3,400	3,404	3,400
adj. *R*^2^	0.050	0.082	0.037	0.081
Weak instrument test (Wald F statistic)			167.6	167.3
Under-identification test (Kleibergen-Paap rk LM statistics):			49.9	49.8

*Robust standard errors in parentheses*;

* *p < 0.10*,

** *p < 0.05*,

*** *p < 0.01*.

**Table 3:** Effects on elderly parents' health.

	**OLS**				**IV**			
	**(1)**	**(2)**	**(3)**	**(4)**	**(5)**	**(6)**	**(7)**	**(8)**
	**Physical health**	**Illness**	**Mental health**	**ADL**	**Physical health**	**Illness**	**Mental health**	**ADL**
**Child quantity and quality**								
Number of children	0.006	−0.015	0.015	−0.035^**^	−0.090^***^	0.492^***^	0.134^**^	0.082^*^
	(0.007)	(0.037)	(0.014)	(0.014)	(0.030)	(0.139)	(0.054)	(0.042)
Primary school	−0.022	−0.548	0.026	0.191	0.003	−0.684	−0.005	0.159
	(0.069)	(0.401)	(0.162)	(0.128)	(0.074)	(0.459)	(0.177)	(0.131)
Lower secondary	−0.032	−0.055	−0.078	0.233^*^	0.023	−0.353	−0.147	0.164
	(0.057)	(0.377)	(0.143)	(0.131)	(0.064)	(0.429)	(0.157)	(0.136)
Upper secondary	0.070	−0.444	−0.009	0.088	0.148^**^	−0.860^*^	−0.106	−0.008
	(0.053)	(0.409)	(0.140)	(0.125)	(0.065)	(0.468)	(0.161)	(0.137)
Vocational training or higher	0.045	−0.355	−0.110	0.209	0.138^**^	−0.850^*^	−0.226	0.094
	(0.056)	(0.415)	(0.149)	(0.130)	(0.068)	(0.475)	(0.169)	(0.142)
**Parent characteristics**								
Living with a son	−0.024	0.174	0.032	0.100^**^	0.032	−0.125	−0.038	0.031
	(0.022)	(0.120)	(0.051)	(0.045)	(0.029)	(0.137)	(0.057)	(0.052)
Living with a daughter	0.026	−0.157	−0.119^*^	−0.018	0.076^**^	−0.420^***^	−0.181^***^	−0.079
	(0.026)	(0.144)	(0.061)	(0.052)	(0.031)	(0.153)	(0.065)	(0.055)
Age	−0.011	0.074	0.116^***^	−0.146^***^	0.018	−0.081	0.079^***^	−0.182^***^
	(0.014)	(0.064)	(0.025)	(0.025)	(0.017)	(0.073)	(0.030)	(0.030)
Age at birth of first child	−0.007^**^	0.030^**^	0.007	−0.002	−0.021^***^	0.104^***^	0.025^***^	0.015^*^
	(0.003)	(0.014)	(0.005)	(0.004)	(0.005)	(0.025)	(0.010)	(0.008)
Male	0.130^***^	−0.934^***^	−0.384^***^	−0.063	0.195^***^	−1.281^***^	−0.465^***^	−0.144^**^
	(0.024)	(0.127)	(0.048)	(0.049)	(0.030)	(0.157)	(0.054)	(0.059)
Married	−0.081^***^	0.307^**^	−0.303^***^	0.101^*^	−0.019	−0.019	−0.380^***^	0.025
	(0.029)	(0.138)	(0.055)	(0.055)	(0.040)	(0.168)	(0.070)	(0.058)
Not working	−0.240^***^	0.349^***^	−0.011	0.426^***^	−0.262^***^	0.465^***^	0.016	0.452^***^
	(0.032)	(0.131)	(0.056)	(0.050)	(0.035)	(0.144)	(0.058)	(0.051)
Kinh (majority)	0.015	−0.649^**^	−0.026	−0.206^**^	−0.022	−0.451	0.020	−0.160
	(0.040)	(0.277)	(0.093)	(0.092)	(0.043)	(0.302)	(0.101)	(0.098)
Religion	0.078^**^	−0.283^*^	−0.002	0.072	0.064^*^	−0.212	0.014	0.088
	(0.034)	(0.165)	(0.053)	(0.058)	(0.034)	(0.169)	(0.054)	(0.059)
Primary	0.018	0.037	−0.037	−0.068	−0.009	0.181	−0.004	−0.035
	(0.035)	(0.166)	(0.055)	(0.061)	(0.038)	(0.179)	(0.058)	(0.063)
Lower secondary	0.090^**^	−0.033	−0.145^**^	−0.051	0.025	0.315	−0.063	0.029
	(0.036)	(0.189)	(0.068)	(0.066)	(0.045)	(0.231)	(0.078)	(0.075)
Upper secondary	0.203^***^	−0.537^**^	−0.138^*^	−0.068	0.095	0.041	−0.003	0.066
	(0.044)	(0.221)	(0.072)	(0.066)	(0.059)	(0.289)	(0.091)	(0.081)
Income (log)	0.382^***^	−1.886^***^	−0.994^***^	−0.606^***^	0.324^***^	−1.582^***^	−0.923^***^	−0.535^***^
	(0.058)	(0.275)	(0.106)	(0.093)	(0.060)	(0.274)	(0.113)	(0.096)
House area (log)	0.046^**^	−0.258^**^	−0.079^**^	−0.016	0.058^**^	−0.319^***^	−0.093^**^	−0.030
	(0.023)	(0.100)	(0.038)	(0.040)	(0.024)	(0.109)	(0.040)	(0.040)
Good housing	0.084^***^	0.071	−0.050	−0.019	0.042	0.294^**^	0.002	0.033
	(0.028)	(0.123)	(0.056)	(0.046)	(0.031)	(0.130)	(0.059)	(0.048)
Constant	2.363^***^	6.599^***^	0.007	5.300^***^	1.836^***^	9.382^***^	0.658	5.945^***^
	(0.478)	(2.287)	(0.868)	(0.790)	(0.529)	(2.391)	(0.919)	(0.879)
*N*	3,602	3,603	3,603	3,603	3,602	3,603	3,603	3,603
adj. *R*^2^	0.140	0.084	0.123	0.156	0.091	0.020	0.101	0.133
Weak instrument test					174.4	175.7	175.7	175.7
Underidentfication test (Kleibergen-Paap rk LM statistic):					53.0	53.2	53.2	53.2

*Robust standard errors in parentheses*;

* *p < 0.10*,

** *p < 0.05*,

*** *p < 0.01*.

In Table 2, the coefficient for the number of children is not statistically significant for all satisfaction measures of elderly parents (columns 1–2). In all cases, the magnitudes of the coefficients for the number of children are very small.

In contrast, we find that the quality of children, as reflected by the highest-educated child's educational level, is associated with higher life satisfaction in parents regarding relationship satisfaction and overall satisfaction. The coefficients for primary and lower secondary schooling are mostly not statistically significant, implying that child education must be at the upper secondary school level or above to affect elderly parents' life satisfaction.

Table 2 also indicates that an elderly married parent has more satisfaction, according to the two satisfaction measures, than a single, divorced, or widowed elderly parent. Male parents are more satisfied with their intra-household relationships than female ones. We find no evidence that age, ethnicity, religion, or age at first birth has any noticeable influence on a parent's life satisfaction. However, there is a strong correlation between wealth, income, and life satisfaction. Older people with higher incomes are happier in both dimensions of life satisfaction. Wealthier households, as proxied by housing quality and house area, have more overall life satisfaction.

Table 3 indicates the impacts of child quantity, quality, and education on elderly parents' health. While the OLS regression shows mostly insignificant coefficients, the IV model indicates that more children lead to worse physical health, more illness issues, ADL issues, and worse mental health. It must be noted that a “positive” coefficient is a good thing for the self-rated physical health measure but a bad thing for the other three health measures.

The quality of children has a beneficial effect on a parents' health with regard to self-reported physical health and illness measures. The effect is only significant at the level of upper secondary school and vocational school or above. However, child quality does not affect ADL or the mental health of parents.

Among other factors, we find a long-term effect of age at first birth on parents' health. Parents who had a child when they were young are more likely to have worse self-rated physical health and more illness, ADL, and mental health issues. Male parents report better health, both physically and mentally, than female parents. We find that married parents have fewer mental health issues than unmarried ones. Non-working older people are more likely to have lower self-rated health and have more illness and ADL issues than working ones. We also find a clear and significant “income effect” on elderly people's health as expected. As proxied by house area, the wealth effect is visible in all measures except the ADL.

While “living with a son” has no impact on parents' health, we find that “living with a daughter” has beneficial impacts on parents' health by boosting self-rated physical health and lessening illness issues and mental health issues. This finding indicates that daughters are more likely to be good health caretakers of their elderly parents. This result can help alleviate a reason for preferring a son over a daughter in Vietnam, i.e., sons are expected to take care of their elderly parents.

### Non-wealthy vs. Wealthy Households

This sub-section divides the sample into two sub-samples: non-wealthy and wealthy households based on household income because their behavior is likely to be affected by wealth. Households with income over 100 million VND (around US$ 4,500) in the past 12 months are classified as wealthy households, while the rest are considered non-wealthy.

Table 4 shows that child quantity leads to lower perceived health and more ADL issues for both sub-groups. However, the effect on illness is significant only for the non-wealthy elderly, while the impact on mental health is significant only for wealthy households. Child quality also has a significant effect in terms of life satisfaction measures in both sub-groups. However, the effects are more substantial for wealthy households than non-wealthy households. This finding indicates that the impact of children's quality is more clearly exhibited in wealthy families than in non-wealthy ones.

**Table 4:** Impacts on parental health and well-being in non-wealthy and wealthy households (IV method).

	**Relationship satisfaction**	**Overall satisfaction**	**Physical health**	**Illness**	**Mental health**	**ADL**
**Non-wealthy households**						
Number of children	−0.037	0.032	−0.084^**^	0.517^***^	0.069	0.109^*^
	(0.043)	(0.052)	(0.038)	(0.163)	(0.066)	(0.057)
Primary school	−0.025	0.047	−0.010	−0.605	0.044	0.184
	(0.092)	(0.108)	(0.079)	(0.495)	(0.177)	(0.150)
Lower secondary	0.094	0.062	0.028	−0.335	−0.072	0.176
	(0.087)	(0.107)	(0.067)	(0.460)	(0.160)	(0.155)
Upper secondary	0.172^**^	0.180	0.133^*^	−0.742	0.015	−0.039
	(0.087)	(0.116)	(0.068)	(0.515)	(0.163)	(0.161)
Vocational training or higher	0.200^**^	0.282^**^	0.141^*^	−0.676	−0.078	0.116
	(0.092)	(0.114)	(0.074)	(0.521)	(0.175)	(0.173)
**Wealthy households**						
Number of children	−0.056	−0.056	−0.122^*^	−0.094	0.472^**^	0.177^*^
	(0.062)	(0.062)	(0.066)	(0.062)	(0.226)	(0.093)
Primary school	0.334	0.334	0.043	0.137	−1.335	−0.323
	(0.253)	(0.253)	(0.284)	(0.226)	(1.039)	(0.488)
Lower secondary	0.497^**^	0.497^**^	0.304	0.066	−0.750	−0.374
	(0.244)	(0.244)	(0.254)	(0.188)	(0.965)	(0.394)
Upper secondary	0.594^**^	0.594^**^	0.436^*^	0.255	−1.625^*^	−0.463
	(0.245)	(0.245)	(0.249)	(0.195)	(0.955)	(0.404)
Vocational training or higher	0.647^***^	0.647^***^	0.495^*^	0.199	−1.661^*^	−0.624
	(0.247)	(0.247)	(0.254)	(0.192)	(0.997)	(0.415)

*Robust standard errors in parentheses*;

* *p < 0.10*,

** *p < 0.05*,

*** *p < 0.01*.

## Discussion

In this study, we attempt to document the impact of adult children on the life satisfaction and health of elderly parents. We find that income and wealth are strong and consistent predictors of older people's life satisfaction and self-perceived health. Among other predictors, the number of children does not affect a parent's life satisfaction but is adversely related to parents' health across a variety of physical and mental health measures.

This finding indicates the negative role of large family size on parents' long-term health. The negative role of family size on self-reported parental health is also found in previous studies such as Islam and Smyth (24) for China and Díaz-Venegas et al. (25) for Mexico.

In addition, our results show the positive effect of child quality, measured by the educational level of the most educated child, on elderly parents' well-being. Parents with better-educated children are more satisfied with their lives and report better health and fewer illness issues. We also find that the impact of children's education on parental health and well-being is larger for wealthy parents, suggesting that child education is more important in wealthy households. These results are in line with Shi (23), who found that the educational level of grown children has a positive effect on elderly parents' quality of life in China.

Our study comes with some limitations. First, our data is cross-sectional survey data in a given year. Having panel data instead of cross-sectional data can undoubtedly help reduce measurement errors and control the impact of omitted variables. Second, the analysis may be improved if we consider more socioeconomic characteristics of the children apart from their education and total number. Third, all health variables are self-reported measures. The findings will be strengthened if we have data on diagnosed health conditions such as body mass index, blood pressure, or cardiovascular health measures. Fourth, more sophisticated life satisfaction measures such as the Satisfaction With Life Scale introduced by Diener et al. (53) or the Riverside Life Satisfaction Scale by Margolis et al. (54) may improve the measurement of life satisfaction in the survey.

## Conclusion

To the best of our knowledge, this is the first study to explore the relationship between quantity and quality of children and the well-being of elderly parents. Our results show that the number of children has an adverse effect on older people's self-reported health and life satisfaction in Vietnam, a large developing country. Meanwhile, parental health and life satisfaction are significantly related to children's education.

The findings of this study provide several practical implications. First, while a quantity-quality tradeoff between the number of children and their human capital is widely accepted in the literature, this paper suggests that this tradeoff can be extended to an intergenerational one between the number of children and parents' health. Second, the paper implies that investment in education for children will have long-lasting impacts on the health and well-being of elderly adults and, therefore, help decrease health care costs for society. Finally, the fact that there is a negative effect of child quantity on parents' health and no effect on parents' life satisfaction helps vindicate the government program for reducing fertility over the last 30 years.

## Data Availability Statement

The data analyzed in this study is subject to the following licenses/restrictions: data available on request due to privacy/ethical restrictions. Requests to access these datasets should be directed to Linh Hoang Vu, vhlinh76@gmail.com.

## Ethics Statement

The studies involving human participants were reviewed and approved by Institute of Social and Medical Studies. The patients/participants provided their written informed consent to participate in this study.

## Author Contributions

LV conceptualized and developed the model, acquired, analyzed, and interpreted the data. LV and TP drafted the article and revised the article. All the authors have approved the submitted version and have agreed to be personally accountable for the author's own contributions and to ensure that questions related to the accuracy or integrity of any part of the work, even ones in which the author was not personally involved.

## Conflict of Interest

The authors declare that the research was conducted in the absence of any commercial or financial relationships that could be construed as a potential conflict of interest. The reviewer TT declared a shared affiliation, with one of the authors, LV, to the handling editor at the time of the review. [2548366].

## Publisher's Note

All claims expressed in this article are solely those of the authors and do not necessarily represent those of their affiliated organizations, or those of the publisher, the editors and the reviewers. Any product that may be evaluated in this article, or claim that may be made by its manufacturer, is not guaranteed or endorsed by the publisher.

## Appendix

**Table A1:** The first stage of the IV model.

	**Number of children**
*Before88^*^ Kinh*	1.395^***^
	(0.098)
Primary school	0.215
	(0.233)
Lower secondary	0.508^**^
	(0.212)
Upper secondary	0.755^***^
	(0.228)
Vocational training or higher	0.835^***^
	(0.226)
**Parent characteristics**
Living with a son	0.509^***^
	(0.062)
Living with a daughter	0.511^***^
	(0.062)
Age	0.072^***^
	(0.004)
Age at birth of the first child	−0.119^***^
	(0.007)
Male	0.654^***^
	(0.062)
Married	0.528^***^
	(0.096)
Not working	−0.214^***^
	(0.065)
Kinh (majority)	−1.604^***^
	(0.180)
Religion	−0.136
	(0.094)
Primary	−0.270^***^
	(0.080)
Lower secondary	−0.698^***^
	(0.084)
Upper secondary	−1.054^***^
	(0.093)
Income (log)	−0.492^***^
	(0.140)
House area (log)	0.101^*^
	(0.055)
Good housing	−0.422^***^
	(0.072)
Constant	2.189^***^
	(0.405)
*N*	3,603
adj. *R*^2^	0.382

*Robust standard errors in parentheses*;

* *p < 0.10*,

** *p < 0.05*,

*** *p < 0.01*.
